# Measures that increase social equality are effective in improving life satisfaction in times of economic crisis

**DOI:** 10.1186/s12889-018-6076-3

**Published:** 2018-11-06

**Authors:** Jocelyne Clench-Aas, Arne Holte

**Affiliations:** 1 Division of Mental and Physical Health, Norwegian Institute of Public Health, PB 4404, Nydalen, NO-0403 Oslo, Norway; 2 Department of Psychology, University of Oslo, Oslo, Norway

**Keywords:** Social inequality, Socioeconomic status, Social gradient, Gini index, Social protection, Financial crisis

## Abstract

**Background:**

The financial crisis of 2008/2009, for some also in 2011, was accompanied by increasing social inequality and unemployment, which strained the welfare generosity systems in many countries. Welfare generosity redistributes internal resources to decrease poverty and increase equal opportunities. This was used by many countries to combat the crisis. We investigated the effects of increased social inequality, unemployment and welfare generosity on life satisfaction (LS) before and after the crisis.

**Methods:**

A representative sample from the European Social Survey (2002 to 2014) with data from 26 countries was used (*N* = 301,559). Time from start of crisis (either 2008 or 2010–2012) was determined separately for each case. LS was measured with a single question with 11 response alternatives. Social inequality was measured using the Gini index. Unemployment was measured by a single question (No/Yes). Welfare generosity was measured using expenditure on social protection (PPS) per inhabitant (Eurostat). Data were analyzed by multilevel analysis and multilevel mediation analysis.

**Results:**

Welfare generosity was associated with decreased levels of social inequality. The negative relationship between social inequality and LS was weakened when controlling for welfare generosity after the financial crisis. This effect of welfare generosity was not seen for the negative impact of unemployment on LS.

**Conclusion:**

The financial crisis stimulated the use of welfare generosity in Europe and strengthened the positive relationship between welfare generosity and LS. Social inequality, unemployment and welfare generosity played significant mediator roles between the crisis and LS, with increased welfare generosity far more strongly associated with increased LS. Measures that increase social equality in a country and thereby increase equal opportunity for all social classes, may be assumed to be effective in improving the general LS of the population in a country in times of economic crisis.

## Background

The financial crisis 2008–2009 provides a unique opportunity to study the relationship between life satisfaction (LS) and socio-economic indicators such as social inequality, unemployment and welfare generosity. In this study we use multilevel analysis of data from around 300,000 respondents in 26 countries to assess how the financial crisis 2008–2009 affected the relationship between social inequality, unemployment, welfare generosity and LS in the European population.

The financial crisis 2008–2009 was a major world event which affected both countries and their inhabitants differently. Poland and Slovakia did not have a recession, whereas other countries had both one recession in 2008 and a second one around 2011. This resulted in large differences in length of the crisis and severity as measured by fall in GDP. The crisis began in the US and had wide repercussions in Europe. Large population groups experienced unemployment, decreased income, loss of benefits, including pensions, and a number of other major life events. The crisis came quickly and was in many cases followed by a long period of recession. Both in the US and in Europe, there were signs of short-term decreases in LS post crisis [1, 2].

Increased financial deregulation following neoliberal reforms in the US, led to increased wealth accumulation in the upper 10% income groups [3–7]. In earlier times, according to classical capitalistic theory, this would lead to increased investments and thus jobs in the commercial sector. This time, however, the increased international growth led to an accumulation of capital in the finance sector. This accumulation of capital, led to an increased distribution of capital in the form of loans to the lower income levels that now had an income level that was insufficient to meet increased expenses [7, 8]. Due to the complexity and magnitude of negative effects on the population of Europe, the crisis made evident the need for social and economic reforms and social investment that specifically targeted the needs of the population [9].

By LS we refer to the cognitive or judgmental component of well-being as opposed to happiness which is the affective component. By welfare generosity we refer to one of several financial measures used by countries to tackle problems following in the wake of financial crises. Welfare generosity implies redistribution of resources through social welfare and social protection of needs including, health, disability, old age, family and unemployment. The aims of generosity measures being to decrease poverty and increase equal opportunity [10]. In response to the 2008–2009 crisis, the European governments varied greatly in their use of welfare generosity measures to decrease the growing social inequality [11]. European countries also varied according to the degree of efficiency of welfare generosity measures [8].

In Europe, the political organization within countries, as defined according to degree of welfare generosity, differs significantly [12]. On the one hand, the Social democratic Welfare State model includes measures to equalize income and educational opportunities. On the other hand, the former Eastern European and Communistic countries have moved from state controlled plan economies that are now in rapid transition into more western norms and in some cases super capitalism. Wealthier countries with a more equal distribution of income and greater economic freedom seem to be associated with a higher degree of happiness and LS [13–15]. LS also seems to be higher in countries with greater political freedom [13].

Social inequality refers to economic differences between the social classes and is often measured using the Gini index. Social inequality has several important dimensions involving differences not only in income and wealth, but also in power, occupational prestige, schooling, ancestry, and ethnicity [8, 16]. The social inequalities that result from economic inequalities reflect the existence of unequal opportunities and rewards for different social groups within a society. Social inequality is not a desirable situation to have for a country [17, 18]. Increased social inequality tends to reduce social mobility, social capital, trust and probably economic growth, and to increase social unrest [16, 19]. Although evidence does not indicate a clear relationship between subjective well-being and social inequality, the impact of social inequality on LS has been little studied [15, 20–25].

Unemployment and income have a large impact on LS and happiness [26, 27]. Unemployment in itself, usually leads to large decreases in well-being that are not only explained by decreased income [26]. The effect of unemployment on cognitive well-being is a sharp negative, followed by an adaptational recovery. This is not so well seen with affective well-being [28].

The combined effect of the crisis and the measures used to meet it, resulted in differences in effects on public health by country [29]. However, in general, the relationship between social inequality, unemployment, welfare generosity and LS in the population has been little studied [30, 31]. Because all these measures are of great significance to the productivity and well-being of populations [32], further knowledge about their relationships and how financial crises may affect them is strongly needed.

### Aims

We have used recommended multilevel techniques [33] to analyze (1) how the financial crisis 2008–2009 influenced social inequality, unemployment, welfare generosity and LS, and (2) how the crisis affected the relationships between the socio-economic measures and LS.

## Methods

We used data from the European Social Survey (ESS). EES is a cross-sectional survey in 36 European countries. The survey has been conducted every 2 years from 2002 through 2016. Data were collected through face-to-face interviews, each lasting approximately 1 h. To ensure that the same methodology is used in all participating countries, ESS has developed standards on sample selection, translation of the questionnaire, data collection and processing, and documentation. The sampling has been conducted with strict random probability methods and the response rate is high in all waves of data collection. Consequently, the data may be considered representative and comparable across nations. The dataset is available on the ESS web page /www.europeansocialsurvey.org. Some of the methods have been described in a previous paper, and in Clench-Aas, J. & Holte, A. Life satisfaction in Europe, Comprehensive effects of social inequality, social mobility and welfare generosity, submitted [1].

We have used data from 2002 through 2014, found on the ESS web page. The questionnaire consists of a core module and two rotating modules. We only used data from the core module. We included data from the 26 countries that had participated in at least three rounds and included the variables of interest. The number of respondents from each country (weighted N) is provided in Table 1. The final sample was *N* = 301,559 (weighted-*N* = 303,410) and consisted of individuals aged 15 and more. Mean age is 48 years, with mean age varying between countries from 44 to 50. There was generally a greater prevalence of women responding with an average of 54%, varying between countries from 48 to 61% women.

**Table 1:** Key features of the financial crisis (FC) for the countries in the survey

	Severity^1^		Financial Crises			Gini index (World Bank Estimate)		Expenditure on social protection PPS in thousand euros		How satisfied with life as a whole		Unemployment	
	W-N	Δ GDP	Rec mnd	Start FC^2^	Start 2nd FC	Pre FC	Post FC	Pre FC	Post FC	Pre FC	Post FC	Pre FC	Post FC
AT	8713	−1.7	21	20082	20113	29.9	30.5	8.4	10.7	7.6	7.3	0.025	0.037
BE	12,575	−2.1	21	20083	20122	29.1	28.2	7.5	9.1	7.4	7.4	0.037	0.037
BG	8324	−6.1	6	20091	0	34.7	35.4	1.4	2.1	4.6	4.6	0.079	0.103
CH	12,333	−1.6	9	20084	0	33.7	32.2	8.8	10.5	8.0	8.1	0.018	0.024
CY	4409	−1.8	54	20091	20113	31.4	32.9	4.4	5.2	7.3	7.0	0.017	0.071
CZ	12,947	−3.7	27	20084	20114	27.2	26.3	3.3	4.6	6.5	6.6	0.033	0.041
DE	20,568	−4.5	12	20082	0	31.9	30.7	7.8	9.4	6.9	7.3	0.066	0.039
DK	10,836	−2.4	24	20083	20113	27.2	29.0	8.3	10.4	8.5	8.4	0.030	0.036
EE	11,391	−9.2	21	20083	20132	34.7	32.8	1.9	3.0	6.1	6.3	0.023	0.035
ES	13,544	−1.6	48	20082	20112	34.1	35.4	4.5	5.8	7.1	7.2	0.041	0.086
FI	14,275	−6.8	30	20081	20122	27.9	27.5	6.7	8.8	8.0	8.0	0.030	0.033
FR	13,004	−1.7	21	20082	20124	30.9	33.2	8.0	9.6	6.4	6.4	0.041	0.052
GB	16,778	−2.3	15	20082	0	35.1	33.9	6.8	7.6	7.1	7.2	0.030	0.039
GR	9759	−4.8	72	20083	0	34.6	34.4	4.0	5.6	6.4	5.9	0.038	0.070
HU	11,697	−4.0	42	20082	20112	28.3	29.3	3.4	3.8	5.6	5.6	0.034	0.059
IE	15,501	−4.1	30	20081	0	33.1	32.4	5.5	7.1	7.6	6.9	0.027	0.095
IT	3696	−2.9	54	20082	20113	34.5	35.2	6.3	7.8	6.7	6.7	0.046	0.097
NL	13,505	−3.2	39	20082	20112	30.2	28.6	8.6	10.4	7.6	7.8	0.021	0.031
NO	11,702	−2.5	18	20091	20102	28.4	25.9	9.2	11.3	7.8	8.0	0.022	0.023
PL	12,430	−0.4	0	0	0	34.4	32.9	3.0		6.7		0.071	0.047
PT	13,718	−2.3	54	20081	20104	38.1	36.2	4.5	5.2	5.7	5.9	0.036	0.074
RU	10,017	−2.0	12	20083	0	41.5	41.3			5.2	5.6	0.016	0.028
SE	13,161	−3.7	15	20081	0	26.6	27.2	8.5	9.4	7.8	7.9	0.033	0.035
SI	9609	−4.4	42	20083	0	28.2	25.0	4.5	5.2	6.8	6.9	0.043	0.046
SK	8931	−9.1	0	0	0	28.3	26.8	3.1		6.3		0.072	0.054
UA	9987	−1.7	21	20082	20113	29.4	25.4			4.4	4.7	0.038	0.050

Severity measured two ways: 1) length of recession in months, and 2) largest decline in consecutive seasonally adjusted GDP per quartile

Blanks indicate missing data for that time period. Poland and Slovakia did not experience a financial crisis

Data were weighted for both design and sample size

*W-N* weighted data, *GDP* Gross Domestic Product, *FC* Financial Crisis

### Measures

The final dependent variable, LS, was assessed by the following item, ‘All things considered, how satisfied are you with your life as a whole nowadays?’. Responses were given on an 11-point scale ranging from 0 to 10 (0 being ‘extremely dissatisfied’ and 10 ‘extremely satisfied’). This one item-scale is one of the most commonly used scales for assessing overall LS and shows moderate to high validity and reliability [34].

#### Independent variables

*Severity of the financial crisis* was measured in two ways: 1) length of recession in months; recession was defined as a minimum of two consecutive quarters with negative change in Gross Development Product (GDP); and 2) largest decline in consecutive seasonally adjusted GDP per quartile for the entire period. The change per quarter in GDP data was extracted from the OECD database for all countries except Russia, Ukraine, Cypress and Bulgaria. For these four countries, the Eurostat database and the World Economic Outlook Database from International Monetary Fund were used [35–37]. A recession was defined for each country as having had a decline in the quarterly seasonally adjusted real GDP for at least two consecutive quarters. The start of the financial crisis was defined as the quarter the decline started for each country. For Portugal, Ireland and Hungary the early recession period in 2007 was not used in defining start point. The length of crises was defined as the total number of months in the entire period (2002–2014) with a recession. Some countries had a second recession after the 2008–2009 financial crisis. A second crisis was defined as a recession with at least two quarters with positive change in GDP between the two episodes that was included in the total length of recession. The summarized data were extracted from the web and were based on data from OECD and Eurostat.

The delay time from the start of the financial crisis to the responses in the questionnaire was determined for each individual by subtracting the individual interview time from the start of the crisis for that country. This resulted, especially for 2008 data, in that the populations from different countries could have different delay times from start of financial crisis. For those countries that had a second recession in 2010/2011, the delay time was adjusted to account for time after the second recession, and was recoded to 1, 2 or 3+ years post-crisis. The delay time was reclassified for this study as pre-crisis, and as post-crisis. The latter was defined as from 1 or more years after the crisis.

*Social inequality* was measured by the Gini index. Data were collected from the World Bank, Development Research Group. Data are based on primary household survey data obtained from government statistical agencies and World Bank country departments (see PovcalNet). Data were collected for each year for each country [38]. The Gini index has a scale of 0 to 100 of 0 represents perfect equality, while an index of 100 implies perfect inequality. Mean for Europe is 32. Mean for USA is 38. The Gini index was matched to the interview date for each subject.

*Welfare generosity* was measured using the social protection data available at Eurostat (ESSPROS). The variable chosen was Expenditure on social protection per inhabitant - PPS per inhabitant (tps00100). Data was missing for Russia and Ukraine. It is defined as “Expenditure on social protection contain: social benefits, which consist of transfers, in cash or in kind, to households and individuals to relieve them of the burden of a defined set of risks or needs; administration costs, which represent the costs charged to the scheme for its management and administration; other expenditure, which consist of miscellaneous expenditure by social protection schemes (payment of property income and other)”. The social protection functions include compensation for sickness/healthcare, disability, old age, survivors, family, unemployment, housing and social exclusion, explained in detail in Eurostat website [39]. For some analyses, the values were described as per 1000 PPS. PPS is the technical term used by Eurostat for the common currency in which national accounts aggregates are expressed when adjusted for price level differences using PPPs. Thus, PPPs can be interpreted as the exchange rate of the PPS against the euro. For practical reasons of readability and interpretation of regression estimates, the values were divided by 1000. The measures used to estimate social protection differ and none are without their limitations. However, using different forms seemed to result in relatively similar results [40–42].

*Unemployment* was measured with one question, “Doing last 7 days: unemployed, actively looking for job”, with response alternatives 0 = No and 1 = Yes.

In addition we adjusted for gender (males = 1; female = 2), age, and age^2^.

#### Variables defining levels

In multilevel analysis, levels are specified prior to the analysis that defines the clusters that the analyses are performed on. For this study, we defined the individual countries as one level. The 26 countries were: Austria (AT), Belgium (BE), Bulgaria (BG), Cyprus (CY), Czech Republic (CZ), Denmark (DK), Estonia (EE), Finland (FI), France (FR), Germany (DE), Greece (GR), Hungary (HU), Ireland (IE), Italy (IT), Netherlands (NL), Norway (NO), Poland (PL), Portugal (PT), Russia (RU), Spain (ES), Slovakia (SK), Slovenia (SI), Sweden (SE), Switzerland (CH), Ukraine (UA), and United Kingdom (GB).

### Statistical analyses

The analyses were conducted using the Statistical Package of the Social Sciences (SPSS), version 24.0. All data were weighted in accordance with the ESS guidelines for the descriptive parts of the study [43]. The primary method of analysis was the multilevel analysis with two levels, the individual (Level 1) and country (Level 2). In SPSS this is done with the module Linear Mixed models [44]. This analysis technique is especially appropriate since there is reason to assume that: 1) values of LS and unemployment within a country have similarities based on social and cultural properties and 2) this method is well suited to data with missing countries for some of the years. The dependent variable was LS. The primary covariates were social inequality, unemployment and welfare generosity, together with gender, age and age^2^. The data were not weighted in these analyses. The intercepts were classified as random. The estimation method was Restricted Maximum Likelihood which is preferred if there are small number of groups within levels. Significance was tested using the LR test statistic (− 2 Log Likelihood) where the difference between the null model and the model under investigation (−2LL null – −2LL level1) varies according to the Chi distribution. The Intraclass correlation (ICC) is calculated as the variance of the intercept (with level indicated)/(residual + variance of intercept). ICC * 100 gives the percent of the variation in the dependent variable attributable to between country variation. Missing data were excluded listwise from the regression analysis.

Mediation analyses were performed within a multilevel context, using the MLMED module for SPSS that works together with the PROCESS module of Hayes [45]. The mediation analyses were performed by entering mediators simultaneously. It was not possible to weight the data in this analysis.

## Results

### The financial crisis

Table 1 shows that the financial crisis varied significantly between countries both in length and severity. While the recession was as short as 6 months in Bulgaria, it lasted for 72 months in Greece. The size of the fall of the GDP growth varied from as little as 1.6 in Spain and Switzerland to 9.2 in Estonia in terms of values for the single quarter with largest decline.

### Life satisfaction

**Fig. 1:**
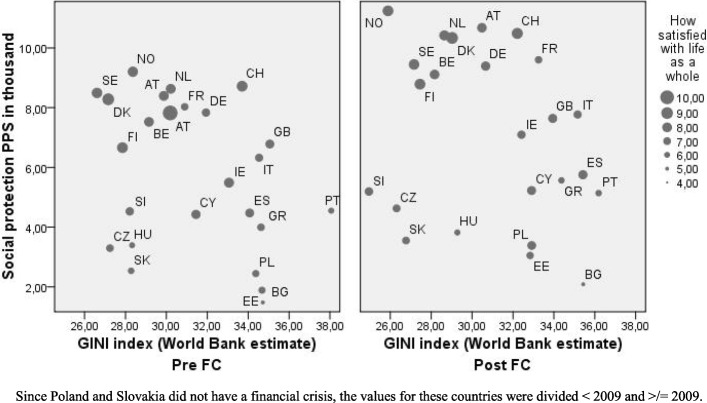
Social inequality (Gini index) and welfare generosity (Social protection) for the period before and after the individually determined Financial Crisis of around 2008

Table 1 and Fig. 1 also shows considerable variation in LS between countries both before and after the crisis. While Ukraine and Bulgaria had the lowest LS scores before the crisis of 4.4 and 4.6, Denmark and Switzerland topped the list with 8.5 and 8.0 points. After the crisis, Ukraine and Bulgaria were still at the bottom with as low LS scores as 4.7 and 4.6, while Denmark and Switzerland again topped the list with scores of 8.4 and 8.1. In addition, Table 1 indicates that the changes from before to after the crisis were in general not large. They varied, however, from a maximum decrease in LS of 9.0% in Ireland to a maximum increase in LS of 7.6% in Russia.

### Social inequality

Table 1 further indicates great variation in social inequality between countries both before and after the crisis. While the Gini index in Denmark and the Czech Republic before the crisis was as low as 27.1, it reached 41.5 in Russia. After the crisis, both Slovenia and Russia had the lowest and the highest index, 25.0 and 41.3, respectively. In some cases, the crisis resulted in increased social inequality, with the greatest seen in France with a 7.6% increase in the Gini index, or as decrease, the greatest being a decrease of − 13.6 and − 11.5% in Ukraine and Slovenia respectively. For Europe as a whole, there was a slight decrease in social inequality the first 2 years after the crisis with a mean pre-crisis of 33.3, to 33.5 at 1 year, then 32.8 at 2 years, followed by a rather sharp increase to 34.3, from 3 years after the crisis (weighted values).

### Welfare generosity

All countries increased their welfare generosity from before to after the crisis, from a mean of 6.3 to progressively 7.8, 8.2 and 8.7 from 1 to 3 years post crisis. Expenditures on social protection increased from a low of 11.1% increase in Sweden to a high of 58.3% in Estonia. The base levels before the crisis, however, were as low as 1500 euros PPS per capita in Bulgaria to a high of 9500 in Norway. Increases in welfare generosity were greatest the first year after the crisis, but continued also after that (data not shown).

### Unemployment

Unemployment levels as measured in this study as the percent of participants actively searching for a job, varied substantially between countries from 1.6 to 7.9% pre-crisis and 2.4–10.3 post-crisis. Levels increased or remained the same from pre to post-crisis for all countries except Germany, Poland and Slovakia. Poland and Slovakia, however, did not have a financial crisis (Table 1).

### Social inequality, unemployment and welfare generosity

Using multilevel mediation analysis (MLMed) (data not shown), we found that the financial crisis led to significant increases in social inequality and unemployment. This contrasts to the fall in social inequality that we found when we only used weighted raw data. Welfare generosity was a significant mediator of the relationship between increasing delay in years after the crisis and both social inequality (standardized within indirect effect = − 0.095, *p* < 0.001) and unemployment (standardized within indirect effect = − 0.011, *p* < 0.001) (*N* = 253,639). This means that as social inequality and unemployment increased, the simultaneous increase in welfare generosity caused the increases in social inequality and unemployment to be significantly less than they would have been otherwise. This mediating effect of welfare generosity was almost 10 times greater for social inequality than for unemployment.

### Social inequality, unemployment, welfare generosity and LS

To assess the difference in importance between social inequality, unemployment, and welfare generosity on LS, we conducted a multilevel analysis by country while controlling for gender, age and age^2^, with both social inequality, unemployment and welfare generosity entered individually and simultaneously.

**Fig. 2:**
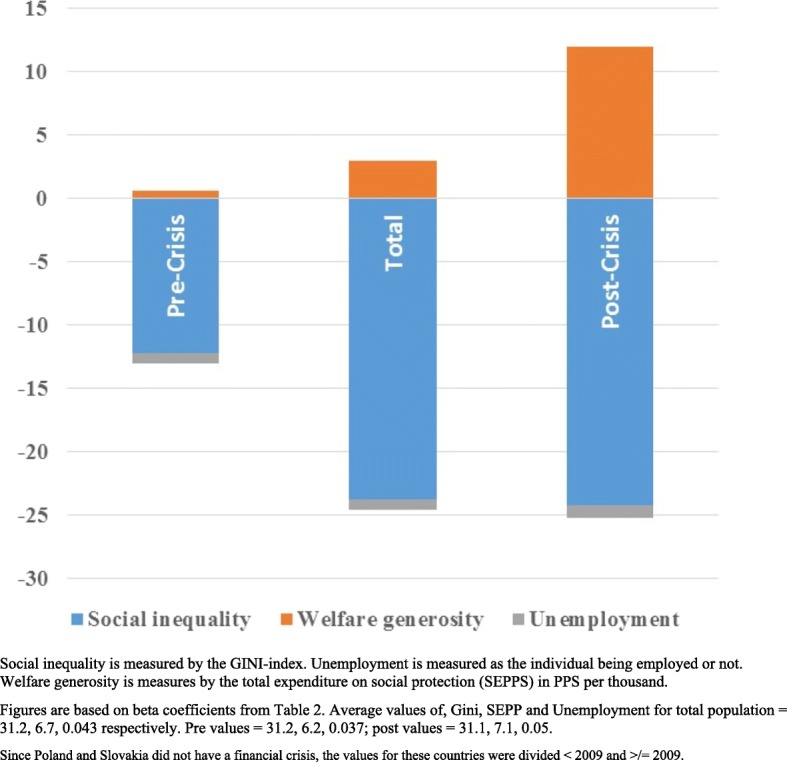
Relative impact of the contextual parameters of social inequality, unemployment and welfare generosity on life satisfaction, for the entire population and entire period, and separated pre and post financial crisis (either 2008–2009 or 2011)

Table 2 reveals reduced LS with increased social inequality. However, when controlling for welfare generosity, the negative effect of social inequality on LS was reduced by 26% whereas there was a positive relationship with welfare generosity. There was a negligible change in the association between LS and unemployment when welfare generosity was added to the regression. As shown in Fig. 2, where the coefficients are combined with the average values of the three measures, social inequality had the greatest impact of the three variables on LS. Examining the results for each of the countries separately (Table 3), shows that there was considerable variation both in the association between LS and social inequality and in the association with unemployment and welfare generosity. Unemployment values, as seen in Fig. 2, did not exhibit a very predominant effect.

**Table 2:** Results (Beta (SE)) of multilevel analysis of satisfaction with life (individual level)

	Satisfaction with life (β/SE/Sig)	Total	Pre FC	Post FC
Social inequality alone	Gini index	− 0.070 (0.003)^***^	− 0.030 (0.006)^***^	− 0.094 (0.008)^***^
	Unemployment	−1.315 (0.019)^***^	− 1.386 (.031)^***^	−1.240 (.024)^***^
Social inequality controlled for welfare generosity (PPS)	Gini index	− 0.052 (.004)^***^	− 0.027 (.007)^***^	− 0.053 (.009)^***^
	Unemployment	−1.360 (.020)^***^	−1.454 (.035)^***^	− 1.274 (.025)^***^
	Total public expenditure social protection PPS	0.030 (006)^***^	0.006(.022)^NS^	0.114 (.012)^***^

All analyses using multilevel (country levels). Russia and Ukraine removed from analysis due to missing data

Baseline included controlled for gender, age, and age^2^, with all three variables entered as fixed

Range: Unemployment = 0–1 (No/Yes); Gini = 23.7–42.3; Social expenditure in PPS per thousand = 1.3–12.0

*FC* Financial Crisis, PPS = Common currency in which national accounts aggregates are adjusted for price level differences using PPPs.

NS = Not Significant; * *p* < 0.05; ** *p* < 0.01; and *** *p* < 0.001

**Table 3:** The results (β/SE/Sig) of separate linear regressions for each country of life satisfaction against first social inequality (Gini index), then welfare generosity (total expenditure on social protection, PPS, in thousand euros), and unemployment while controlling for gender, age and age^2^

Country	Social inequality				Welfare generosity				Unemployment			
	Unstandardized		Standardized		Unstandardized		Standardized		Unstandardized		Standardized	
	B	Std. Error	Beta	Sig.	B	Std. Error	Beta	Sig.	B	Std. Error	Beta	Sig.
AT	− 0.067	0.069	− 0.010	0.335	0.020	0.037	0.006	0.597	− 1.220	0.137	− 0.096	0.000
BE	−0.014	0.017	−0.007	0.421	0.040	0.019	0.020	0.038	− 1.159	0.086	−0.121	0.000
BG	0.114	0.028	0.043	0.000	0.014	0.074	0.002	0.853	− 1.427	0.094	−0.165	0.000
CH	−0.061	0.015	−0.035	0.000	0.017	0.016	0.011	0.277	− 1.447	0.104	−0.125	0.000
CY	−0.115	0.024	−0.072	0.000	−0.395	0.062	−0.096	0.000	− 1.093	0.140	−0.118	0.000
CZ	−0.120	0.036	−0.029	0.001	0.024	0.034	0.007	0.490	− 1.469	0.097	−0.133	0.000
DE	−0.299	0.017	−0.119	0.000	0.292	0.016	0.126	0.000	− 2.042	0.073	−0.193	0.000
DK	−0.011	0.013	−0.008	0.377	−0.013	0.013	−0.010	0.326	−0.858	0.081	−0.102	0.000
EE	−0.144	0.019	−0.071	0.000	0.255	0.038	0.062	0.000	− 1.513	0.114	−0.122	0.000
ES	−0.075	0.016	−0.044	0.000	−0.050	0.035	−0.014	0.145	− 1.222	0.073	−0.156	0.000
FI	−0.060	0.037	−0.014	0.104	0.002	0.012	0.001	0.874	−0.942	0.073	−0.108	0.000
FR	−0.014	0.016	−0.007	0.401	0.058	0.021	0.024	0.006	− 1.690	0.097	−0.150	0.000
GB	−0.053	0.017	−0.027	0.002	0.105	0.036	0.027	0.003	− 1.097	0.094	−0.102	0.000
GR	0.019	0.049	0.004	0.699	−0.279	0.028	−0.099	0.000	− 1.036	0.099	− 0.106	0.000
HU	0.058	0.018	0.032	0.001	0.111	0.116	0.010	0.337	− 1.442	0.118	−0.121	0.000
IE	0.170	0.021	0.065	0.000	−0.398	0.020	−0.159	0.000	− 1.515	0.068	−0.180	0.000
IT	0.110	0.137	0.013	0.422	−0.107	0.048	−0.037	0.024	− 1.091	0.153	−0.119	0.000
NL	−0.073	0.013	−0.050	0.000	0.079	0.015	0.051	0.000	−0.989	0.084	−0.102	0.000
NO	−0.057	0.008	−0.065	0.000	0.105	0.014	0.074	0.000	− 1.135	0.104	−0.100	0.000
PL	−0.367	0.022	−0.146	0.000	0.598	0.048	0.129	0.000	− 1.286	0.092	−0.124	0.000
PT	−0.144	0.018	−0.072	0.000	0.420	0.053	0.075	0.000	−0.624	0.080	−0.069	0.000
RU	−0.164	0.093	−0.017	0.078					−0.950	0.150	−0.062	0.000
SE	0.039	0.044	0.008	0.370	0.026	0.030	0.008	0.385	− 1.234	0.083	−0.131	0.000
SI	−0.034	0.009	−0.039	0.000	−0.057	0.065	−0.010	0.385	− 1.092	0.109	−0.101	0.000
SK	−0.394	0.028	−0.153	0.000	0.741	0.043	0.183	0.000	− 1.590	0.104	−0.163	0.000
UA	−0.113	0.012	−0.092	0.000					− 1.106	0.117	−0.093	0.000

### Social inequality, unemployment, welfare generosity, LS and the financial crisis

When comparing these relationships before and after the financial crisis, the findings were even stronger. In the pre-crisis situation, the negative effect of increased social inequality on LS was not significantly changed when we controlled for welfare generosity. Welfare generosity in itself had a non-significant relationship with LS. In the post-crisis situation, the negative effect of increased social inequality on LS was decreased by 44% when welfare generosity was introduced. There was a strong positive relationship between LS and welfare generosity (*p* < 0.001), that showed a major increase as opposed to the pre situation where it was not even significant. The association of unemployment with LS showed a slight weakening post-crisis. In Europe (Fig. 2), the crisis was associated with an increase in the negative impact of social inequality. The impact of welfare generosity in the form of social protection measures post-crisis on the life satisfaction of the population was increased. As may also be seen in Fig. 2, the association with unemployment was relatively stable.

### Mediator analysis

**Fig. 3:**
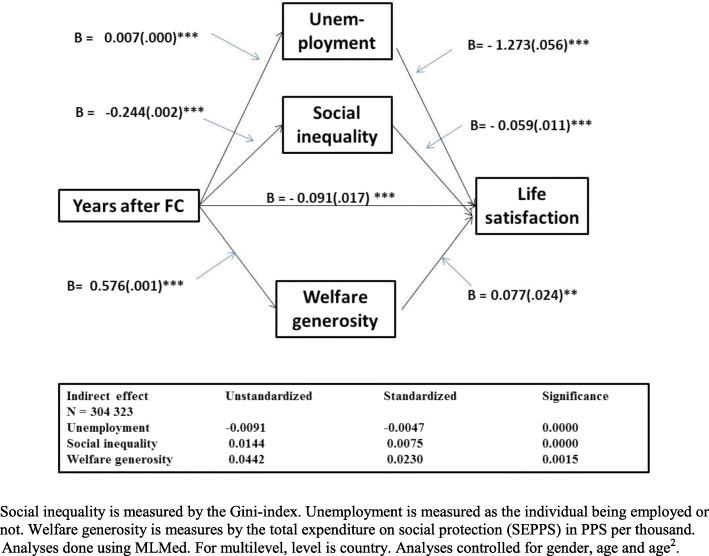
Mediator analysis of the association between delay in years after either 2008 or 2011 financial crisis determined individually for each country, and life satisfaction with social inequality, unemployment and welfare generosity

Mediator analysis (Fig. 3) revealed a significant mediator role for all three characteristics, social inequality, unemployment and welfare generosity, in the relationship between years after the financial crisis and LS. Both the stimulating role of the financial crisis in increasing welfare generosity and the subsequent positive association between welfare generosity and LS were confirmed. There was a significant slight decrease in social inequality as a result of the crisis when unemployment and welfare generosity were accounted for. The results also confirmed that higher social inequality is associated with lower LS. Increase in unemployment was associated with substantially lower LS. Examining the standardized indirect effect, revealed that the indirect positive effect of welfare generosity was almost 3 to 5 times greater than the indirect effects of either social inequality or unemployment. The effect of social inequality was slightly greater than the effect of unemployment. The direct effect of the financial crisis on LS was negative.

### Multilevel analysis

The significance of using multilevel analysis was tested by the Chi^2^ test with and without entering the country level. When country level was not included AIC was 595,617. When country level was included AIC was 585,294. This resulted in a ΔAIC of 10,323 (*p* < 0.000). Intraclass correlation (ICC) was 19.8% of the variation explained.

## Discussion

In this study we utilized the 2008–2009 financial crisis in Europe combined with multilevel analysis and multilevel mediation analysis to examine how changes in social inequality and unemployment from before to after the crisis, and the social protection measures taken to meet these challenges, changed the relationships between the socio-economic measures and LS in the population. Our findings adds to previous knowledge in several ways.

First, we observed great variation between countries in how the financial crisis affected countries in length, severity, GDP, social inequality, unemployment, how they used social protection measures to counter its effects, and in LS. This is consistent with the description given by many after the financial crisis [11, 29, 46–48].

Second and surprisingly, we found that the changes in LS were generally small. The high stability in LS is consistent with our previous finding that, initially when the crisis arrived, a fall in LS occurred. However, this fall was only of short duration after which LS recovered to its original level, in some countries even better [1]. Such a decline and rather rapid recovery has previously been observed in the USA [2, 28]. We believe that this pattern reflects the more general idea that LS is determined more by the social and educational capital inherent in a population than in transitory economic fluctuations [13, 14].

Third, we observed a slight decline in social inequality the first 2 years after the crisis, followed by a sharp increase 3 years after the crisis. Unemployment increased as well, although remaining unaffected in some countries. The short-term decline in social inequality has been reported earlier, and may possibly be explained by the richest being hardest hit in the crisis. However, the long term trends have been towards increasing social inequality, not in the least in the recovery period after the crisis where the rich showed the strongest recovery [19, 49].

Fourth, we found that all countries increased their welfare generosity, some even substantially. This increase was most intensive the first year, but continued also in the following years. This is consistent with the notion that increased income redistribution, in the form of welfare generosity is a policy measure that may reduce both social inequality and unemployment. Other effective measures include increasing the efficiency of welfare generosity [8]. Our study did in fact corroborate that the increase in welfare generosity was accompanied with a corresponding decrease in primarily social inequality but also unemployment. This is consistent with the ideas presented by Leoni, where the concept of social investment, which includes both social protection measures and social activation measures such as education, and active labor market policies, have been promoted as necessary policy measures to counter the problems following from the financial crisis [9, 50].

Fifth, social inequality had a negative effect on LS. When we controlled for the effect of welfare generosity on LS over the whole period, the negative effect of social inequality on LS was reduced by 26%. This is in contrast to a clear ambiguity in results in many previous studies [15, 20–25]. However, the change in the association between unemployment and LS was only negligible.

Sixth, the relationships between social inequality, unemployment and welfare generosity, respectively, and LS changed considerably from before to after the crisis. Whereas before the crisis welfare generosity had little impact on the negative effect of increased social inequality on LS, post-crisis adjusting for welfare generosity decreased the negative impact of social inequality by 44%. The positive relationship between welfare generosity and LS while not significant pre-crisis, exhibited a major positive impact post-crisis. This shows that there is no simple relationship between social inequality, unemployment, welfare generosity and LS, and that these relationships change with changing socio-economic conditions. Most importantly, it indicates that the positive effect of welfare generosity on LS reduces the negative impact of social inequality. There is some support for this in another study also based on ESS data [15]. Since there is no way of having a control situation without the effect of a financial crisis, these changes are only indicative. It cannot be ruled out that other changes in the time period not related to the financial crisis may have influenced the findings.

## Strengths and limitations

One main strength of this study is the large sample size obtained by ESS and their use of methodological standards at all stages in the process. This makes the data representative and ideal for comparative and cross-national analyses. The ESS team is working continuously to ensure high validity and reliability of the questionnaire and data collected. They use strict randomized probability sampling and the questionnaire is well tested and translated according to ESS protocols. Another strength is the use of multilevel analyses and multilevel mediation analysis. Multilevel analysis allows testing for fundamental relationships in individuals while accounting for the importance of the influence of the social context underlying population groups, and especially countries. This makes it possible to examine well-being in individuals while accounting for constraints imposed by the political organization of countries, in for example social inequality, unemployment and welfare generosity. Even though we have used terms like affect, impact and influence, the cross-sectional nature of the survey limits the possibility to draw causal conclusions from the findings. Data were collected through self-report. Response bias may be therefore be present. This primarily concerns the objective data (e.g. education, income), not the subjective measures (e.g. LS). Since the financial crisis was global, there is no control group in this kind of study. Each country’s pre-crisis situation was used as a control for the post-situation. Although, it might be suggested to use countries like Slovakia and Poland that did not experience a financial crisis as a control, this is not possible because as transition countries, they had a special situation with strong growth that inhibited the development of a financial crisis.

## Conclusion

The political desirability of examining progression in a country by examining well-being as opposed to GDP was put forth in 2009 by Sarkozy, resulting in the Stiglitz report [51]. That report emphasized that high satisfaction with life in the population is a value in itself and argued that countries should pursue LS rather than purely productivity [51]. In addition, LS has also been shown to contribute to political stability, high productivity and effectiveness, good health, better physical and mental health, longevity and improved interrelationships between individuals in a country [52–57]. Strange then, that the relationship between LS and socioeconomic indicators such as social inequality, unemployment and welfare generosity has been little studied.

In this paper we have asked whether social inequality, unemployment and welfare generosity were driving forces in determining life satisfaction before and after the 2008 financial crisis in Europe? Our answers are: Yes, the financial crisis both stimulated the use of welfare generosity in Europe and strengthened the positive relationship between welfare generosity and LS. Yes, social inequality, unemployment and welfare generosity played significant mediator roles between the crisis and LS. Yes, increased social inequality was associated with decreased LS, but increased welfare generosity was far more strongly associated with increased LS and countered the negative effect on social inequality. In conclusion, measures that reduce social inequality in a country and thereby increase equal opportunity for all social classes, may be assumed to be effective in improving the general LS of the population in a country.

## Abbreviations

AIC: Akaike Information Criteria
ESS: European Social Survey
GDP: Gross Domestic Product
ICC: Intraclass Correlation
ISCED: International Standard Classification of Education
ISCO88: International Standard Classification of Occupation
LR: Likelihood ratio
LS: Life Satisfaction
OECD: Organization for Economic Cooperation and Development
PPP: Purchasing Power Parities
PPS: Common currency in which national accounts aggregates are adjusted for price level differences using PPPs.
SE: Standard Error
W-N: Weighted sample size

## Countries

AT: Austria
BE: Belgium
BG: Bulgaria
CH: Switzerland
CY: Cyprus
CZ: Czech Republic
DE: Germany
DK: Denmark
EE: Estonia
ES: Spain
FI: Finland
FR: France
GB: United Kingdom
GR: Greece
HU: Hungary
IE: Ireland
IT: Italy
NL: Netherlands
NO: Norway
PL: Poland
PT: Portugal
RU: Russia
SE: Sweden
SI: Slovenia
SK: Slovakia
UA: Ukraine
